# Source-Free Domain-Adaptive Semi-Supervised Learning for Object Detection in CCTV Images

**DOI:** 10.3390/s26010045

**Published:** 2025-12-20

**Authors:** Hyejin Shin, Gye-Young Kim

**Affiliations:** Department of AI·SW Convergence, Soongsil University, Seoul 06978, Republic of Korea; hjshinhjshin@soongsil.ac.kr

**Keywords:** CCTV, object detection, domain adaptation, source-free, semi-supervised learning

## Abstract

Current object detection methods deployed in closed-circuit television (CCTV) systems experience substantial performance degradation due to domain gaps between training datasets and real-world environments. At the same time, increasing privacy concerns and stricter personal data regulations limit the reuse or distribution of source-domain data, highlighting the need for source-free learning. To address these challenges, we propose a stable and effective source-free semi-supervised domain adaptation framework based on the Mean Teacher paradigm. The method integrates three key components: (1) pseudo-label fusion, which combines predictions from weakly and strongly augmented views to generate more reliable pseudo-labels; (2) static adversarial regularization (SAR), which replaces dynamic discriminator optimization with a frozen adversarial head to provide a stable domain-invariance constraint; and (3) a time-varying exponential weighting strategy that balances the contributions of labeled and unlabeled target data throughout training. We evaluate the method on four benchmark scenarios: Cityscapes, Foggy Cityscapes, Sim10k, and a real-world CCTV dataset. The experimental results demonstrate that the proposed method improves mAP@0.5 by an average of 7.2% over existing methods and achieves a 6.8% gain in a low-label setting with only 2% labeled target data. Under challenging domain shifts such as clear-to-foggy adaptation and synthetic-to-real transfer, our method yields an average improvement of 5.4%, confirming its effectiveness and practical relevance for real-world CCTV object detection under domain shift and privacy constraints.

## 1. Introduction

Closed-circuit television (CCTV) systems are widely used in video surveillance and play a crucial role in enhancing situational awareness and response. Their integration with emerging technologies such as artificial intelligence (AI), facial recognition, and big data analytics has substantially expanded their capabilities and societal impact [1,2]. Modern CCTV-based perception systems rely heavily on deep learning models trained on large-scale datasets. However, in real-world deployments, these models frequently encounter environments that differ considerably from their training distributions. This discrepancy, known as the domain gap or domain shift, arises from variations in weather, lighting, image resolution, camera characteristics, or scene geometry. When the gap is large, object detection accuracy can deteriorate sharply, limiting the reliability and practical utility of deployed systems [3]. Domain-adaptive object detection (DAOD) aims to bridge this gap by aligning features or representations across domains so that a model trained on a source domain can perform robustly in a target domain. Prior work has explored a broad range of domain adaptation strategies, including adversarial feature alignment [4,5], distribution discrepancy minimization [6], data-level adaptation via image style transfer [7,8], pseudo-label-based self-training under teacher–student and consistency regularization [9,10,11,12,13,14,15], pseudo-label quality enhancement for cross-domain or adverse-weather adaptation [16,17,18,19,20], and source-free teacher–student adaptation without accessing source data [21,22,23]. In addition, instance-level, spatial alignment [24,25,26], and curriculum-based optimization strategies [27] have been used. These approaches have shown promising results, particularly when both source and target data can be leveraged during training. However, traditional domain adaptation methods, which typically involve repeated retraining or accumulation of new training data, come with substantial costs related to data storage and computational resources. In addition, rising concerns about data privacy and the increasing enforcement of data protection regulations make it difficult to reuse previously collected training datasets [28]. In addition, legal and ethical constraints—such as data ownership issues or privacy legislation—may entirely prohibit access to historical training data. In such contexts, source-free learning has emerged as a promising alternative, enabling adaptation to new domains using only a pre-trained model and unlabeled target data, without any access to source-domain data [29]. Most existing source-free learning approaches rely exclusively on unlabeled target data for adaptation [30], which introduces major challenges, including training instability and reduced detection accuracy [21]. These issues become especially problematic when the domain gap is large, severely limiting the practical utility of such methods. While various recent techniques have been introduced to address these shortcomings, a major challenge remains: the absence of reliable supervision in the form of ground-truth labels.

**Figure 1:**
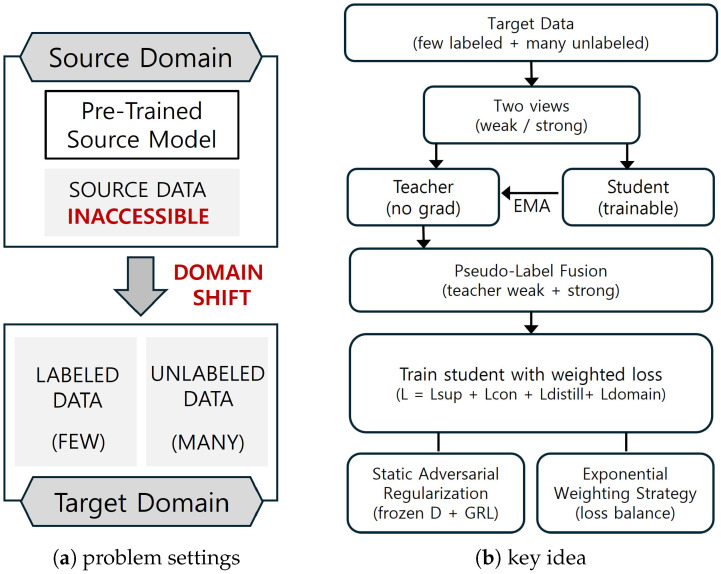
Concept diagram. (**a**) Problem setting: source data are inaccessible under domain shift with few labeled and many unlabeled target samples. (**b**) Key idea: Mean Teacher with pseudo-label fusion, static adversarial regularization, and exponential loss weighting.

Figure 1 summarizes this problem setting and the key ideas of our approach. As shown in Figure 1a, we consider a source-free semi-supervised adaptation scenario where source-domain data are inaccessible under domain shift, and only a few labeled target samples are available together with abundant unlabeled target data. Figure 1b highlights our key ideas: pseudo-label fusion combines weakly and strongly augmented views of the same target sample to generate more robust and consistent pseudo-labels for unlabeled-target supervision; static adversarial regularization (SAR) freezes the discriminator and uses a gradient reversal layer (GRL) to impose a stationary confusion constraint, mitigating source-specific overfitting and promoting domain-agnostic features; and an exponential weighting strategy applies a time-varying schedule to balance the learning contributions of labeled and unlabeled target data, stabilizing training and improving adaptation effectiveness.

Motivated by this setting, we propose a source-free domain adaptation approach that enables stable training using only target-domain data, without requiring access to any source data. Our method is designed to reduce the domain gap and enhance detection accuracy and reliability by utilizing a small set of labeled target samples in conjunction with a large pool of unlabeled target data. Built on the Mean Teacher framework [9]—which consists of a student-teacher model pair, our approach enables effective self-training and adaptation within the target domain. The key components of our method include the following:

Pseudo-Label Fusion, which combines weakly and strongly augmented samples to generate more robust and consistent pseudo-labels.
Static Adversarial Regularization (SAR), which freezes the discriminator and uses gradient reversal layer (GRL) to impose a stationary confusion constraint, mitigating source overfitting and yielding domain-agnostic features.
Exponential Weighting Strategy, a time-varying scheme that balances the learning contributions of labeled and unlabeled data to stabilize training and improve effectiveness.

The main contributions of this paper are summarized as follows:

- Performance improvement in source-free learning via pseudo-label fusion: Enhances detection accuracy by producing more reliable pseudo-labels through the fusion of predictions from weakly and strongly augmented views of the same input.
- Promoting cross-domain generalization via SAR: Replaces the dynamic min-max update with a frozen, randomly initialized discriminator and trains only the backbone through a gradient-reversal pathway. This turns the adversarial head into a stationary regularizer that imposes a fixed confusion objective, curbs source-specific overfitting, stabilizes training, and yields domain-agnostic features that transfer better to the target domain without accessing source data.
- Balancing learning by label availability: Employs a time-varying exponential weighting that adjusts the contributions of labeled and unlabeled data during training, promoting stable and efficient learning.
- A practical framework for domain-adaptive, source-free semi-supervised learning: We propose a practical framework tailored for real-time object detection scenarios, such as CCTV. To isolate and evaluate the contributions of the proposed adaptation method under a consistent detector architecture, we employ YOLOv7 [31] as the base detector throughout all experiments. This choice keeps the detector architecture consistent across all experiments, helping us attribute performance gains to our adaptation components rather than to changes in the detector itself.

## 2. Related Work

### 2.1. Domain-Adaptive Object Detection

Domain adaptation (DA) [32] aims to improve model performance in a target domain by narrowing the domain gap between source and target data, thereby facilitating the transfer of knowledge learned from the source domain. Domain-adaptive object detection (DAOD) extends this concept specifically to object detection, where the objective is to detect and localize objects in target-domain images. The primary challenge in DAOD lies in overcoming visual discrepancies between domains—such as differences in weather, lighting conditions, and image resolution. While conventional DA techniques can be adapted for detection tasks, the added complexity of localization (i.e., predicting bounding boxes) presents unique challenges.

Existing approaches to DAOD can be broadly categorized based on the availability of labeled data:

- Fully supervised adaptation: Both source and target domains have labeled data.
- Semi-supervised adaptation: The source domain is labeled and the target domain is unlabeled.
- Source-free unsupervised adaptation: Only unlabeled target-domain data are used, and no access to source data is required.

Fully supervised DA for object detection: This approach utilizes labeled data from both the source and target domains, allowing for direct supervision and domain alignment based on target annotations. For example, IA-YOLO [7] integrates a fully differentiable image processing (DIP) module into the YOLO architecture to suppress weather-induced distortions. A CNN-based parameter predictor (CNN-PP) dynamically tunes DIP hyperparameters based on input conditions, ensuring reliable detection across varying weather scenarios. Similarly, PICA [24] proposes a point-wise instance alignment strategy for few-shot adaptation using limited target labels. To mitigate label noise in background regions (ROIs), it employs a moving-average-based centroid method and restricts alignment to the classification branch, leading to performance gains.

Semi-supervised DA for object detection: Semi-supervised approaches rely on labeled source data and unlabeled target data, focusing on transferring knowledge while handling the absence of target labels. Probabilistic Teacher [18], for instance, models uncertainty in target predictions and enforces consistency without relying on hard confidence thresholds, thereby improving both classification and localization, while Adaptive Teacher [19] employs a teacher model to generate pseudo-labels, utilizing domain-adversarial learning and weak-to-strong augmentation for better generalization. To address pseudo-label noise and foreground-background overfitting, Unbiased Teacher [11] adopts a progressive teacher–student learning strategy. Further, CMT [15] extracts object-level features from pseudo-labels and enhances them via contrastive learning without needing ground truth, while ConfMix [20] further refines adaptation by blending confidently pseudo-labeled regions with source images. Additionally, SSDA-YOLO [16] minimizes domain shifts using artificially generated images for both domains, while GCHQ [17] focuses on generating high-quality pseudo-labels under adverse weather conditions.

Source-free DA for object detection: This emerging approach exclusively uses unlabeled target-domain data during training, omitting source data entirely. Its practicality lies in situations where source data cannot be shared due to privacy, ownership, or legal constraints. However, the lack of source supervision introduces significant challenges, such as training instability and performance degradation under large domain shifts. Methods like LODS [8] attempt to bypass style-specific discrepancies, while IRG [25] improves contrastive learning through an instance relation graph network. MemCLR [33] leverages a cross-attention transformer-based memory module to store and retrieve prototype representations, enabling strong instance-level learning. EBCDet [27] applies an energy-based curriculum learning strategy to incrementally train the model while minimizing noise from incorrect pseudo-labels caused by domain shifts.

Each of these approaches comes with trade-offs:

- Fully supervised methods offer high accuracy but require extensive annotations.
- Semi-supervised methods reduce annotation needs but may suffer from noisy pseudo-labels and training instability.
- Source-free methods are highly practical but struggle with stable, high-performance adaptation due to the absence of source data.

In this study, we present a novel source-free domain-adaptive object detection framework that addresses these limitations by significantly enhancing training stability and adaptation performance while using only a small amount of labeled target data and not requiring any source-domain data.

### 2.2. Mean Teacher

The Mean Teacher framework [9] is a training methodology that utilizes both student and teacher models to enhance generalization, particularly in domain adaptation tasks. The central principle of this approach is to ensure consistency between the predictions made by the student and teacher models, which improves robustness and stability during training. The student model updates its weights by minimizing the loss based on labeled data, while the teacher model’s weights are updated indirectly via an exponential moving average (EMA) of the student’s parameters. This mechanism provides more stable and consistent predictions.

During training, both the student and teacher models generate predictions for the same input data. The discrepancy between these predictions is computed as a consistency loss, which the student model aims to minimize. The teacher model’s weights are updated using the EMA of the student’s parameters, which is formulated as follows:

(1) $$\theta_{t}^{\prime }=\alpha \theta_{t-1}^{\prime }+\left(1-\alpha \right)\theta_{t}$$

where $\theta_{t-1}^{\prime }$ and $\theta_{t}^{\prime }$ represent the teacher model’s weights from the previous and current steps, respectively, $\theta_{t}$ is the current weight of the student model, and α is the EMA momentum parameter.

EMA gives more weight to recent updates while exponentially diminishing the influence of older updates, allowing the teacher model to capture stable changes over time. This approach helps the teacher model to progressively learn generalized features, making the Mean Teacher framework particularly effective at leveraging unlabeled data to improve generalization and robustness, and also ensures stable training, even in the presence of noisy or inaccurate labels.

Recent studies have built upon the Mean Teacher framework to further enhance training stability and the reliability of pseudo-labels. For example, UMT [14] addresses structural biases by incorporating cross-domain distillation and pixel-level adaptation techniques. Humble Teacher [13] introduces soft pseudo-labels, enabling the student model to capture more diverse information while dynamically adjusting the reliability of pseudo-labels to reduce learning errors, while Soft Teacher [12] applies weighted bounding box classification losses based on the teacher model’s predictions and incorporates box jittering to improve the accuracy of pseudo-box regression. PETS [21] introduces a periodically exchanging teacher–student framework to mitigate model collapse caused by domain shifts, thereby enhancing the stability of the student model, and DRU [22] actively manages the learning and updating process between the student and teacher using a dynamic retraining–updating strategy and reduces the impact of inaccurate pseudo-labels by utilizing historical student loss information. Lastly, SF-YOLO [23] develops a target-specific augmentation module that generates augmented images tailored to the target domain for training the student model. Additionally, to prevent drift caused by pseudo-labels, it stabilizes the student model by updating it through the teacher model’s EMA.

## 3. Proposed Method

**Figure 2:**
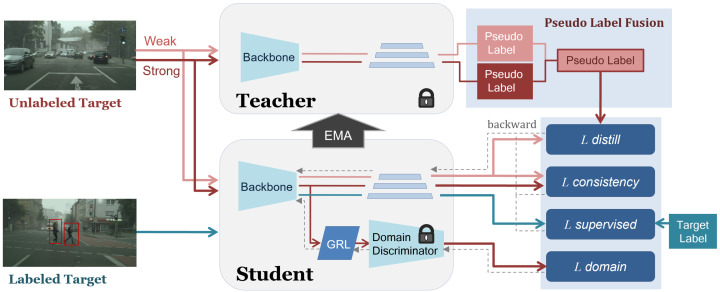
Overview of the proposed method architecture.

This study introduces a domain-adaptive, source-free, semi-supervised learning approach aimed at improving object detection performance in the target domain without relying on source data. Our method builds upon the Mean Teacher framework, where the student model learns from high-confidence pseudo-labels generated by the teacher model, using target-domain data. This approach effectively mitigates domain shifts, enhancing recognition accuracy in the target domain without the need for source data. To further improve model robustness and stability during domain adaptation, we incorporate several key techniques: pseudo-label fusion using weak–strong augmented data, SAR for stable adversarial regularization, and a time-varying (epoch-scheduled) exponential weighting that adjusts the contributions of labeled and unlabeled data. The overall architecture of the proposed method is depicted in Figure 2.

### 3.1. Problem Formulation

In this section, we formalize the source-free semi-supervised domain adaptation (SF-SSDA) setting for object detection. Let $X\subset R^{H\times W\times 3}$ denote the input image space and Y denote the label space comprising bounding box coordinates and class categories.

- Notations: We consider two distinct domains: a source domain $D_{S}$ and a target domain $D_{T}$.
  · The Source Domain is fully labeled and denoted as $D_{S}={\left\{\left(x_{s}^{i},y_{s}^{i}\right)\right\}}_{i=1}^{N_{s}}$, where $N_{s}$ is the number of source samples.
  · The Target Domain is partially labeled. It consists of a small set of labeled samples $D_{T}^{l}={\left\{\left(x_{t}^{i},y_{t}^{i}\right)\right\}}_{i=1}^{N_{l}}$ and a significantly larger set of unlabeled samples $D_{T}^{u}={\left\{x_{t}^{j}\right\}}_{j=1}^{N_{u}}$, where $N_{l}\ll N_{u}$.
- Model Architecture: The object detector $F_{\theta }$ is defined as a composition of a feature extractor (backbone) $G_{\theta_{G}}:X\to Z$ and a detection head $H_{\theta_{H}}:Z\to Y$, such that $F_{\theta }\left(x\right)=H_{\theta_{H}}\left(G_{\theta_{G}}\left(x\right)\right)$. Here, Z represents the high-dimensional feature space.
- Problem Settings and Assumptions: Our framework operates under the following specific assumptions designed to reflect real-world privacy constraints in CCTV surveillance:
  1. Source-Free Constraint: The source dataset $D_{S}$ is accessible only during the pre-training phase. During the target adaptation phase, access to $D_{S}$ is strictly prohibited due to privacy or data retention policies.
  2. Closed-set Detection. The class set is assumed to be shared across domains, while the data distributions differ due to environmental variations (e.g., weather, illumination, and camera characteristics).
  3. Domain Shift: There exists a significant distribution shift between the source and target domains, i.e., $P_{S}\left(x,y\right)\neq P_{T}\left(x,y\right)$, which degrades the performance of the model trained solely on $D_{S}$ when applied to $D_{T}$.
  4. Label Scarcity: The target domain relies primarily on unsupervised learning via $D_{T}^{u}$, supplemented by sparse supervision from $D_{T}^{l}$.
- Learning Objective (Overview): Based on these definitions, the target adaptation aims to optimize the student parameters $\theta_{S}$ without source data. The optimization objective is a combination of (i) supervised detection loss on the labeled target set $D_{T}^{l}$, (ii) consistency- and pseudo-label-based distillation loss on the unlabeled target set $D_{T}^{u}$, and (iii) a domain confusion regularization term (SAR), modulated by a time-varying weighting strategy described in the subsequent sections.

These definitions and assumptions serve as the theoretical foundation for our proposed framework described in the subsequent sections.

### 3.2. Mean-Teacher-Based Source-Free Semi-Supervised Learning

The student and teacher models in our framework share the same architecture and are initialized with identical parameters. The teacher model generates pseudo-labels by making predictions on target-domain images. Unlike the student model, the teacher is not updated through direct backpropagation; instead, its parameters are updated using an EMA of the student model’s parameters. The student model is trained on a small set of labeled target images and a larger set of unlabeled target images. Throughout training, the quality of the pseudo-labels produced by the teacher model improves gradually, thereby enhancing the student’s learning effectiveness.

### 3.3. Pre-Training on the Source Domain

In domain adaptation, pre-training on the source domain is an essential step before adaptation of the target domain, particularly in source-free learning scenarios where direct access to source data during adaptation is restricted. Only the pre-trained model parameters are retained and used for subsequent training on the target domain; however, models pre-trained solely on source data often overfit to source-specific artifacts, which degrades generalization to the target domain. To address this, we recast adversarial learning as static adversarial regularization (SAR)—a stationary regularizer instantiated via a frozen discriminator and a gradient reversal layer (GRL). The resulting pre-trained weights serve as a stable, domain-agnostic initialization for both the student and the teacher models during target-domain training.

### 3.4. Static Adversarial Regularization

To improve training stability in the source-free semi-supervised adaptation framework and to suppress overfitting to domain-specific artifacts, we introduce static adversarial regularization (SAR). In contrast to domain-adversarial neural networks (DANN) [4], which adopt dynamic adversarial learning via a minimax game between a feature extractor and a trainable discriminator, SAR uses a randomly initialized and frozen discriminator as a static regularizer. By removing discriminator updates, it avoids training instability that can occur in dynamic adversarial learning, especially when adaptation involves noisy pseudo-labels and only a small labeled target set is available.

- Simplified explanation: Dynamic adversarial learning updates both the backbone and the discriminator, and the training behavior can become sensitive to their relative learning speeds, potentially resulting in unstable gradients. SAR removes this source of instability by freezing the discriminator and using it only as a fixed regularization anchor. Through a gradient reversal layer (GRL), the backbone receives a stationary gradient signal from the fixed discriminator, discouraging domain-specific feature cues while keeping optimization stable.
- Motivation (SAR vs. dynamic DANN): In source-free semi-supervised adaptation, unlabeled target learning relies on pseudo-labels and thus inevitably includes noise, while only a small portion of target samples is labeled. Under such conditions, minimax optimization can be fragile: the discriminator may become overly confident or poorly calibrated, and the resulting gradients can destabilize adaptation. SAR addresses this by eliminating discriminator updates entirely (i.e., the discriminator is frozen and excluded from optimization), thus acting as a stable regularization term that suppresses domain-discriminative activations in the backbone without requiring dynamic adversarial updates.
- Mechanism: Let $f=G_{\theta_{G}}\left(x\right)$ denote backbone features extracted from an input image *x*, and let $D_{\phi }$ be a domain discriminator attached to *f* through a GRL. Domain labels are defined as $y_{d}\in \left\{0,1\right\}$ (source =0, target =1). In SAR, $D_{\phi }$ is randomly initialized once and kept frozen throughout both source pre-training and target adaptation; thus, gradients from the SAR loss update only the backbone (and detector) parameters, while ϕ is never updated. We note that $D_{\phi }$ is never optimized; SAR backpropagates gradients only to the backbone through GRL, encouraging the backbone to learn less domain-discriminative representations.
- Rationale for Random Initialization (Random-Projection Perspective): In SAR, the discriminator is not trained to become an optimal domain classifier; instead, it serves as a fixed projection head that provides a stationary adversarial signal through the GRL. Because $D_{\phi }$ is never optimized, SAR eliminates the min–max coupling in dynamic adversarial learning and yields stable, deterministic gradients to the backbone. Although randomly initialized, a fixed discriminator defines random separating directions in feature space; making features less predictive under these fixed projections suppresses domain-discriminative cues across many directions, acting as a form of random-projection regularization.
- Formal objective: Using binary_cross_entropy_with_logits as the domain loss ($L_{\mathrm{bce}}$), SAR is applied in both stages as follows. For source pre-training, we define:
  (2) $$L_{\mathrm{SAR}}^{\left(s\right)}=E_{x∼D_{S}}\left[L_{\mathrm{bce}}\left(D_{\phi }\left(\mathrm{GRL}\left(G_{\theta_{G}}\left(x\right)\right)\right),\;0\right)\right],$$
  and for target adaptation, we define:
  (3) $$L_{\mathrm{SAR}}^{\left(t\right)}=E_{x∼D_{T}^{u}}\left[L_{\mathrm{bce}}\left(D_{\phi }\left(\mathrm{GRL}\left(G_{\theta_{G}}\left(x\right)\right)\right),\;1\right)\right],$$
  Because GRL reverses gradients before they reach the backbone, optimizing these terms provides a regularizing update that discourages domain-discriminative cues in the feature extractor while keeping the discriminator fixed.
- Implementation note: The discriminator parameters are excluded from the optimizer and remain unchanged; SAR is used solely to provide a stationary regularization signal to the backbone via GRL.

### 3.5. Pseudo-Label Fusion

In most existing approaches, pseudo-labels are generated by feeding weakly augmented images into the teacher model. However, this strategy often lacks prediction diversity and robustness, making it vulnerable to variations in object location, scale, or shape. To address these limitations, we propose a more robust pseudo-labeling strategy that leverages both weakly and strongly augmented versions of unlabeled target-domain images.

For each target image, we apply independent weak and strong augmentations, and the teacher model generates predictions on both versions. Predictions with low objectness scores are filtered out to exclude unreliable candidates. Rather than relying on non-maximum suppression (NMS), which retains only the highest-scoring bounding box and suppresses others, we employ weighted box fusion (WBF) [34] for each set of predictions, which aggregates all overlapping boxes by computing a confidence-weighted average of their coordinates. This results in more stable and informative detections—an especially valuable property in source-free scenarios where ground-truth annotations are unavailable. Once WBF is applied separately to both the weakly and strongly augmented predictions, the resulting two sets of fused boxes are further combined using Soft-NMS [35]. Unlike standard NMS, which aggressively removes overlapping detections, Soft-NMS reduces their confidence scores based on overlap, allowing for a more nuanced and flexible merging process, which helps to preserve potentially valid detections that might otherwise be discarded.

Our proposed technique improves the quality and reliability of pseudo-labels, enhances training stability, and significantly boosts model generalization—particularly in domain adaptation settings characterized by high variability and uncertainty.

### 3.6. Target-Domain Training

The teacher and student models share the same network architecture and are initialized with parameters pre-trained on the source domain. Training is then conducted exclusively on target-domain images, with no access to source data. The teacher model generates pseudo-labels for unlabeled target images by performing predictions on both weakly and strongly augmented versions of the inputs. Importantly, the teacher model is not updated directly during training; instead, its parameters are updated indirectly through an EMA of the student model’s weights. The student model is trained using a combination of a small set of labeled target data and a larger set of unlabeled target data. Its learning process is guided by four distinct loss functions: supervised loss, consistency loss, distillation loss, and domain loss. Each of these components contributes to improving different aspects of the model’s performance—ensuring label accuracy, maintaining prediction stability, transferring knowledge from the teacher model, and reducing domain-specific discrepancies, respectively. After each training iteration, the student model’s updated parameters are used to gradually refine the teacher model via EMA, enabling the teacher to evolve with the student and continuously adapt to the target domain.

#### 3.6.1. Supervised Loss

The student model generates predictions on labeled target-domain images, and the supervised loss is computed based on the discrepancy between these predictions and the corresponding ground-truth annotations. This loss function follows the standard formulation used in the YOLOv7 framework and is defined in Equation (4):

(4) $$L_{\mathrm{supervised}}=L_{\mathrm{cls}}+L_{\mathrm{box}}+L_{\mathrm{conf}}$$

where

· $L_{\mathrm{cls}}$ is the classification loss, which evaluates whether the predicted class matches the ground-truth label using BCE;
· $L_{\mathrm{box}}$ is the bounding box regression loss, computed using the complete intersection over union (CIoU) between predicted and ground-truth boxes;
· $L_{\mathrm{conf}}$ is the objectness loss, which determines the presence of an object in each grid cell and is also calculated using BCE.

**Figure 3:**
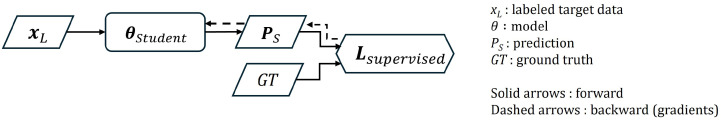
Supervised loss diagram.

The supervised loss branch is illustrated in Figure 3. Labeled target data is fed into the student model to produce predictions. The supervised loss between this prediction and the ground-truth label is then computed, and the model is trained by minimizing this loss.

#### 3.6.2. Consistency Loss

Consistency loss is designed to enforce alignment between the predictions generated from weakly and strongly augmented versions of the same unlabeled target image. To strengthen this consistency, the strong augmentation includes a horizontal flip of the weakly augmented image [10]. The total consistency loss is the sum of classification and bounding box consistency losses, as shown in Equation (5):

(5) $$L_{\mathrm{consistency}}=L_{\mathrm{cons}\_\mathrm{cls}}+L_{\mathrm{cons}\_\mathrm{box}}$$

The classification consistency loss $\left(L_{\mathrm{cons}\_\mathrm{cls}}\right)$ is defined as the average of two one-way consistency losses: (1) weak-to-strong, and (2) strong-to-weak predictions, as shown in Equation (6):

(6) $$L_{\mathrm{cons}\_\mathrm{cls}}=\frac{L_{\mathrm{cls}}^{w\to s}+L_{\mathrm{cls}}^{s\to w}}{2}$$

where the one-way classification consistency losses are as defined in Equations (7) and (8).

(7) $$L_{\mathrm{cls}}^{w\to s}=\frac{1}{bs}\sum_{i=1}^{bs}\frac{1}{M}\sum_{j=1}^{M}L_{\mathrm{cons}}\left(w_{i}^{\left(j\right)},s_{i}^{\left(j\right)}\right)$$

(8) $$L_{\mathrm{cls}}^{s\to w}=\frac{1}{bs}\sum_{i=1}^{bs}\frac{1}{M}\sum_{j=1}^{M}L_{\mathrm{cons}}\left(s_{i}^{\left(j\right)},w_{i}^{\left(j\right)}\right)$$

where

· bs is the batch size;
· *M* is the number of predicted bounding boxes per image;
· $w_{i}^{\left(j\right)}$ and $s_{i}^{\left(j\right)}$ represent the logits of the *j*-th bounding box in the *i*-th image under weak and strong augmentations, respectively.

The loss between these logits is computed using the Kullback–Leibler (KL) divergence:

(9) $$\begin{array}{cc} L_{\mathrm{cons}}\left(w_{i},s_{i}\right)\; & =D_{\mathrm{KL}}\left(\mathrm{softmax}\left(w_{i}\right)\;∥\;\mathrm{softmax}\left(s_{i}\right)\right)\\ & =\sum_{c=1}^{nc}\mathrm{softmax}{\left(w_{i}\right)}_{c}\log\frac{\mathrm{softmax}{\left(w_{i}\right)}_{c}}{\mathrm{softmax}{\left(s_{i}\right)}_{c}} \end{array}$$

where $w_{i,c}$ and $s_{i,c}$ are the predicted class probabilities for class *c*. These logits are formally defined as follows:

(10) $$w_{i},s_{i}\in R^{M\times nc},\;\mathrm{where}\;w_{i}=f_{\theta }\left(x_{w_{i}}^{u}\right),\;s_{i}=f_{\theta }\left(x_{s_{i}}^{u}\right)$$

where $x_{w}^{u}$ and $x_{s}^{u}$ denote the strongly and weakly augmented versions of the same unlabeled image $x^{u}$, and $f_{\theta }\left(\cdot \right)$ represents the model output (logits). *M* is the number of predicted boxes per image, and nc is the number of classes. In addition, $w_{i}$ and $s_{i}$ represent the class logits for the *i*-th image under strong and weak augmentations, respectively. The bounding box consistency loss ($L_{cons\_box}$) is calculated as the mean squared error (L2 loss) between the bounding box predictions from weak and strong augmentations:

(11) $$L_{\mathrm{cons}\text{\_}\mathrm{box}}=\frac{1}{4}\left(L_{x}+L_{y}+L_{w}+L_{h}\right)$$

Each component of this loss is computed as in Equation (12).

(12) $$\begin{array}{cc} L_{x} & =\frac{1}{bs}\sum_{i=1}^{bs}\frac{1}{M}\sum_{j=1}^{M}{\left(x_{w_{i}}^{\left(j\right)}+x_{s_{i}}^{\left(j\right)}-1\right)}^{2}\\ L_{y} & =\frac{1}{bs}\sum_{i=1}^{bs}\frac{1}{M}\sum_{j=1}^{M}{\left(y_{w_{i}}^{\left(j\right)}-y_{s_{i}}^{\left(j\right)}\right)}^{2}\\ L_{w} & =\frac{1}{bs}\sum_{i=1}^{bs}\frac{1}{M}\sum_{j=1}^{M}{\left(w_{w_{i}}^{\left(j\right)}-w_{s_{i}}^{\left(j\right)}\right)}^{2}\\ L_{h} & =\frac{1}{bs}\sum_{i=1}^{bs}\frac{1}{M}\sum_{j=1}^{M}{\left(h_{w_{i}}^{\left(j\right)}-h_{s_{i}}^{\left(j\right)}\right)}^{2} \end{array}$$

where x,y denote the bounding box center coordinates, and w,h represent the width and height, respectively. Let $w_{i}^{\left(j\right)}=\left(x_{w},y_{w},w_{w},h_{w}\right)$ be the *j*-th bounding box in the *i*-th image under strong augmentation, and $s_{i}^{\left(j\right)}=\left(x_{s},y_{s},w_{s},h_{s}\right)$ be the corresponding prediction under weak augmentation.

In particular, the horizontal component $L_{x}$ incorporates horizontal flipping by encouraging $x_{s}+x_{w}\approx 1$, thereby promoting feature-level symmetry and regularizing the detector.

**Figure 4:**
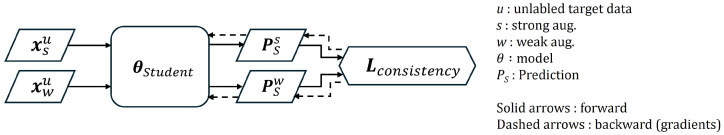
Consistency loss diagram.

The consistency loss branch is illustrated in Figure 4. The strongly and weakly augmented versions of an unlabeled image, $x_{s}^{u}$ and $x_{w}^{u}$, are fed into the student model to obtain the corresponding predictions $P_{s}^{s}$ and $P_{w}^{s}$. The consistency between these two predictions is then measured using the equations defined above and optimized as the consistency loss to update the parameters of the student model.

#### 3.6.3. Distillation Loss

**Figure 5:**
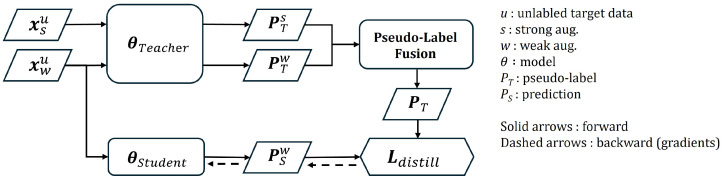
Distillation loss diagram.

Distillation loss measures the discrepancy between the pseudo-labels generated by the teacher model and the predictions made by the student model, when both are fed augmented versions of the same unlabeled target image. This loss encourages the student model to refine its predictions on unlabeled data by learning from the teacher’s high-confidence pseudo-labels. Structurally, the distillation loss adopts the same formulation as the supervised loss described in Equation (4), combining classification, bounding-box regression, and objectness components. By leveraging this loss, the knowledge encoded in the teacher’s pseudo-labels is effectively transferred to the student model. The distillation loss branch is illustrated in Figure 5. For an unlabeled image, the teacher model receives its weakly and strongly augmented versions and generates predictions, which are then integrated into a soft target, $P_{T}$, through pseudo-label fusion. The student’s prediction, $P_{w}^{S}$, based on the weakly-augmented unlabeled image, is trained to match $P_{T}$ via $L_{distill}$, and the gradients are propagated only to the student.

#### 3.6.4. Domain Loss

**Figure 6:**
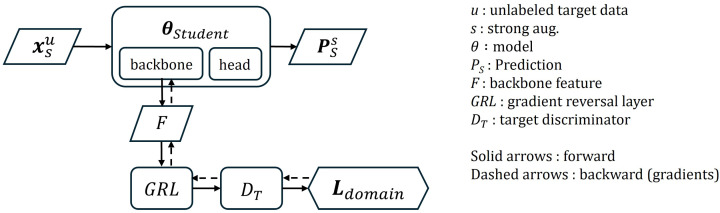
Domain loss diagram.

Domain loss for the target-domain training stage is implemented using the static adversarial regularization (SAR) loss for target adaptation, $L_{\mathrm{SAR}}^{\left(t\right)}$, which was formally defined in Equation (3). This loss is applied exclusively to unlabeled target-domain images ($x_{t}$). As defined in Section 3.4, this loss measures how effectively the feature extractor confuses the fixed target discriminator ($D_{t}$) into classifying target features ($F_{t}$) as the opposite ‘source’ (label 0). This is in order to aligns to align the target feature distribution with the domain-agnostic feature space being learned. During backpropagation, the GRL reverses the gradient direction from the discriminator, forcing the feature extractor to maximize the domain loss and thereby causing $D_{t}$ to misclassify the target features. By satisfying this static constraint, the feature extractor is guided to minimize domain-specific biases and generate domain-invariant representations. The domain loss branch is illustrated in Figure 6. Unlabeled target data with strong augmentation ($x_{s}^{u}$) are fed into the student model, and the features extracted from the student model’s backbone are passed through a GRL to the domain discriminator. The discriminator is trained to classify whether the input features originate from the source domain or the target domain. During backpropagation, the GRL reverses the sign of the gradients from the discriminator. As a result, the backbone parameters are updated in a direction that makes it increasingly difficult for the discriminator to distinguish between domains, effectively maximizing the domain loss from the backbone’s perspective.

### 3.7. Labeled and Unlabeled Data Weight Adjustment

The final loss function used in this paper is defined in Equation (13).

(13) $$L_{\mathrm{total}}\left(t\right)=\alpha \left(t\right)L_{\sup}+\frac{\beta (t)}{2}L_{\mathrm{cons}}+\frac{\beta (t)}{2}L_{\mathrm{distill}}+L_{\mathrm{domain}}$$

In the early stages of training, a relatively high weight, α(t) is assigned to the supervised loss $L_{sup}$, computed from labeled data, while lower weights, β(t), are assigned to the consistency loss ($L_{cons}$) and distillation loss ($L_{distill}$), both derived from unlabeled data. This strategy helps stabilize training in the early phase and facilitates faster initial convergence. As training progresses, α(t) is gradually decreased and β(t) is increased, progressively enhancing the contribution of unlabeled data to the learning process. The adjustment of these weights follows exponential decay functions, as shown in Equations (14) and (15):

(14) $$\alpha \left(t\right)=\alpha_{\mathrm{start}}\cdot {\left(\frac{\alpha_{\mathrm{end}}}{\alpha_{\mathrm{start}}}\right)}^{t/T}$$

(15) $$\beta \left(t\right)=\beta_{\mathrm{start}}\cdot {\left(\frac{\beta_{\mathrm{end}}}{\beta_{\mathrm{start}}}\right)}^{t/T}$$

where *t* denotes the current training epoch and *T* represents the total number of training epochs. $\alpha_{start}$ and $\beta_{start}$ represent the initial values of α and β, respectively, while $\alpha_{end}$ and $\beta_{end}$ represent their final values. This gradual weight adjustment strategy encourages the model to begin training in a stable manner by relying on trustworthy supervised signals. Once training stabilizes, the model increasingly leverages unlabeled signals more effectively. Consequently, this approach enhances both the training balance and the model’s generalization performance.

The proposed training procedure is outlined in Algorithm 1. The implementation is available at https://github.com/hjshine/SFDASSL (accessed on 10 December 2025). The repository provides the code used in our experiments and will be maintained with minor updates for documentation and usability.
**Algorithm 1** Semi-supervised Source-free Domain-Adaptive Training.**Input:**
    Labeled data $D_{T}=\left\{X_{T},Y_{T}\right\}$
*(Labeled target domain data: 0.5—10%)*
    Unlabeled data $D_{U}=\left\{X_{U}\right\}$
*(Unlabeled target domain data)*
    $X_{U\_W}$: Weakly augmented version of $X_{U}$
    $X_{U\_S}$: Strongly augmented version of $X_{U}$
    Student model $\theta_{S}$, Teacher model $\theta_{T}=\theta_{S}$, EMA decay α, Optimizer η
**Output:**
    Trained student model $\theta_{S}$
1:**for** each epoch **do**2:      ALPHA, BETA ← AdjustWeightsExponential()3:      **for** each mini-batch $\left(X_{T},Y_{T}\right)\in D_{T}$ and $X_{U}\in D_{U}$ **do**4:            $P_{T\_U\_W}\leftarrow \mathrm{TeacherModel}\left(\theta_{T},X_{U\_W}\right)$5:            $P_{T\_U\_S}\leftarrow \mathrm{TeacherModel}\left(\theta_{T},X_{U\_S}\right)$6:            $P_{T}\leftarrow \mathrm{PseudoLabelFusion}\left(P_{T\_U\_W},P_{T\_U\_S}\right)$7:            $P_{S}\leftarrow \mathrm{StudentModel}\left(\theta_{S},X_{T}\right)$8:            $P_{S\_U\_W}\leftarrow \mathrm{StudentModel}\left(\theta_{S},X_{U\_W}\right)$9:            $P_{S\_U\_S}\leftarrow \mathrm{StudentModel}\left(\theta_{S},X_{U\_S}\right)$10:          $F_{T}\leftarrow \mathrm{GRL}\left(F\right)$                                       ▹*F: backbone features, $F_{T}$: grad reverse features*11:          ${\mathrm{Loss}}_{sup}\leftarrow \mathrm{SupervisedLoss}\left(P_{S},Y_{T}\right)$12:          ${\mathrm{Loss}}_{cons}\leftarrow \mathrm{ConsistencyLoss}\left(P_{S\_U\_W},P_{S\_U\_S}\right)$13:          ${\mathrm{Loss}}_{distill}\leftarrow \mathrm{DistillationLoss}\left(P_{S\_U\_W},P_{T}\right)$14:          ${\mathrm{Loss}}_{domain}\leftarrow \mathrm{DomainLoss}\left(F_{T}\right)$15:          $\mathrm{TotalLoss}\leftarrow \mathrm{ALPHA}\cdot {\mathrm{Loss}}_{sup}+\left(\mathrm{BETA}/2\right)\cdot {\mathrm{Loss}}_{cons}+\left(\mathrm{BETA}/2\right)\cdot {\mathrm{Loss}}_{distill}+{\mathrm{Loss}}_{domain}$16:          $\theta_{S}\leftarrow \theta_{S}-\eta \cdot \nabla_{\theta_{S}}\mathrm{TotalLoss}$                                        ▹*Update the student model*17:          $\theta_{T}\leftarrow \alpha \cdot \theta_{T}+\left(1-\alpha \right)\cdot \theta_{S}$                                         ▹*Update the teacher model*18:     **end for**19:**end for**20:**return** $\theta_{S}$


## 4. Experiments

### 4.1. Configuration

The experimental setup used in this study is as follows. The operating system is Ubuntu 20.04, and the GPU used is an NVIDIA GeForce RTX 4090.

#### 4.1.1. Datasets

**Figure 7:**
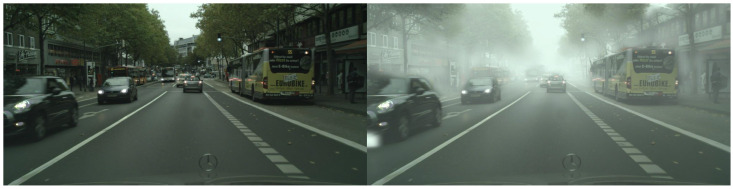
Example images from the Cityscapes and Foggy Cityscapes datasets (**Left**: Cityscapes; **Right**: Foggy Cityscapes).

To evaluate the performance of the proposed method, three datasets were utilized in the experiments:

Cityscapes and Foggy Cityscapes datasets [36]: These datasets are widely used for domain adaptation tasks [37]. The Cityscapes dataset consists of 3475 images, with 2975 used for training and 500 for testing. As Foggy Cityscapes is generated by applying simulated fog to the Cityscapes images, contains the same number of images. In the experiments, Cityscapes is treated as the source domain to evaluate domain adaptation performance from clear weather to foggy weather conditions. For the target domain, consistent with prior works [16,20,22,23], we use Foggy Cityscapes with the highest fog density (0.02), as illustrated in Figure 7.

**Figure 8:**
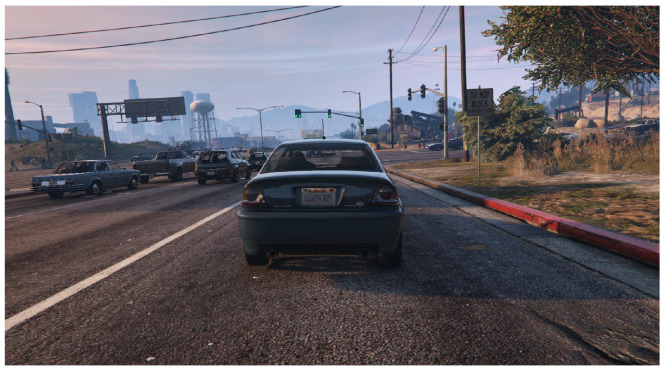
Example images from the Sim10k dataset.

Sim10k Dataset [38]: This is a synthetic image dataset that focuses on vehicle instances. An example image is shown in Figure 8. In our study, Sim10k and Cityscapes serve as the source and target domain, respectively, allowing us to evaluate domain adaptation performance from synthetic to real-world images. The Sim10k dataset consists of 10,000 images, with 9000 used for training, 500 for testing, and 500 for validation.

**Figure 9:**
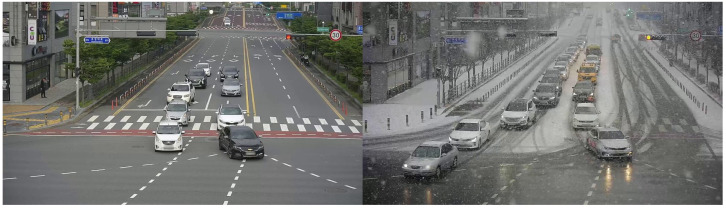
CCTV dataset images (**Left:** Clear weather, **Right**: Snowy weather).

CCTV Traffic Video Dataset (Urban Roads) [39]: This dataset was collected from CCTV footage for traffic-related applications. For domain adaptation, we first selected a specific region with snowy-weather data. Then, clear-weather and snowy-weather images were separately extracted from daytime video footage. Examples of the extracted images are shown in Figure 9. In this experiment, clear-weather data serve as the source domain and snowy-weather data are the target domain. The source domain consists of 3000 images, with 2550 used for training and the remaining 450 for testing. The target domain contains 352 images, of which 300 are used for training and 52 for testing. This dataset configuration is used to evaluate domain adaptation performance from clear- to snowy-weather conditions.

#### 4.1.2. Implementation Details

The experiments were conducted using the YOLOv7 architecture. We set the input image resolution to 960×960 pixels to effectively detect small and distant objects inherent in CCTV surveillance footage. This higher resolution is particularly beneficial in CCTV imagery where targets occupy only a small fraction of the frame due to a wide field of view (FoV) and long viewing distances. This setting follows the experimental protocols established in recent domain adaptation studies such as SSDA-YOLO [16], GCHQ [17], and SF-YOLO [23]. Weakly augmented images were generated using Mosaic augmentation, while strongly augmented images were created by applying a series of transformations—ColorJitter, HueSaturation, CLAHE, GaussNoise, and HorizontalFlip—on top of the weakly augmented inputs.

For optimization, we employed stochastic gradient descent (SGD). Regarding optimizer settings, we adopted transfer-learning hyperparameters commonly used in surveillance object detection [40]: a momentum of 0.843 and a weight decay of 0.00036. Unlike the default hyperparameters commonly used for training from scratch, these values were selected to stabilize fine-tuning and reduce catastrophic forgetting during adaptation. The initial learning rate (lr0) was set to 0.0016 with a final cosine decay factor (lrf) of 0.1, using a conservative fine-tuning schedule to mitigate the impact of noisy pseudo-labels in the early stages of source-free adaptation [23]. For pseudo-label generation, we set the IoU threshold to 0.3 and adopted a conservative confidence threshold (τ) of 0.75 to suppress false positives, consistent with conservative filtering commonly used in teacher–student detectors (e.g., Unbiased Teacher [11]).

The EMA rates used to update the teacher model varied depending on the domain adaptation task. An EMA rate of 0.9996 was applied for both Cityscapes → Foggy Cityscapes and Sim10k → Cityscapes, consistent with the recommended settings in the original Mean Teacher framework [9]. We chose this higher rate for large-scale benchmarks to provide a more stable teacher and reduce sensitivity to short-term noise in pseudo-labels. In contrast, a lower rate of 0.96 was used for the smaller CCTV Clear → Snowy scenario to allow faster teacher adaptation (i.e., less lag between teacher and student) under conditions of limited data and stronger domain shift.

All three domain scenarios involved pre-training on source-domain data for 300 epochs, followed by adaptation training for 60 epochs in each of the Cityscapes → Foggy Cityscapes and Clear → Snowy (CCTV) settings, and 100 epochs in the Sim10k → Cityscapes setting. These epoch numbers were selected empirically based on convergence behavior observed on the validation set and overall training stability, rather than adopting a fixed default. In particular, the adaptation stage was kept comparatively shorter to reach a stable performance plateau while limiting prolonged training that can amplify early pseudo-label noise and overfit to target-specific artifacts in source-free adaptation.

To investigate the influence of labeled target data on performance, experiments were conducted using different proportions of labeled target images: 0.5%, 1%, 2%, 5%, and 10%. The model performance was quantitatively evaluated using standard object detection metrics, including precision, recall, mAP@0.5, and mAP@0.5:0.95.

### 4.2. Results on the Cityscapes to Foggy Cityscapes Dataset

Following initialization of both the teacher and student models using a pre-trained model on the source domain (Cityscapes), training was conducted on the target domain (Foggy Cityscapes) according to the proposed method. The results are presented in Table 1 which compares performance before and after domain adaptation using varying proportions of labeled target data: 0.5%, 1%, 2%, 5%, and 10%. Performance was evaluated on both the source (Cityscapes) and target (Foggy Cityscapes) domains using four metrics: precision, recall, mAP@0.5, and mAP@0.5:0.95. For each metric, the best result on the target domain is shown in bold, and the best result on the source domain is underlined. In addition, changes relative to the baseline (i.e., before target-domain training) are shown in parentheses as the percentage increases or decreases.

A detailed analysis of the results in Table 1 reveals that the detection performance on the Foggy Cityscapes dataset steadily improves as the proportion of labeled data in the target domain increases. Notably, when 10% of the target data are labeled, the mAP@0.5 on the target domain reaches 60.9, which is a 10.0% improvement over the baseline performance prior to adaptation. In contrast, the mAP@0.5 on the source domain (Cityscapes) decreases by 7.6%, yielding a score of 63.6. A similar trend is observed for mAP@0.5:0.95, with the target domain improving by 6.4% to 39.4, while the source domain declines by 5.1% to 40.5.

We note that the trend across label ratios may not be perfectly monotonic because the effective supervision changes during adaptation: as the labeled target ratio increases, the contribution of target supervised loss becomes stronger and shifts the optimization focus toward the target distribution. Moreover, in low-label regimes, higher sampling variance (e.g., the class composition and difficulty of the labeled subset) and pseudo-label noise can lead to saturation or small fluctuations rather than strictly monotonic gains.

These findings indicate that the performance gains on the target domain outweigh the losses on the source domain. This suggests that the proposed method adapts effectively to the target domain while keeping the source-domain degradation moderate. Furthermore, the results show that effective domain adaptation is achievable even with limited labeled target data, and that catastrophic forgetting is relatively well controlled.

Table 2 provides a quantitative comparison of domain adaptation performance from Cityscapes to Foggy Cityscapes against various existing methods. The evaluation is based on class-wise average precision (AP) and the mean AP at IoU 0.5 (mAP@0.5). The compared methods are categorized into four types according to their training strategies:

- Type A: Few-shot supervised learning methods that utilize labeled source data and a small amount of labeled target data.
- Type B: Semi-supervised learning methods using labeled source data and unlabeled target data.
- Type C: Source-free unsupervised learning methods relying solely on unlabeled target data, without access to source data.
- Type D: The proposed source-free semi-supervised learning method, which leverages a small amount of labeled target data along with a larger portion of unlabeled target data, without any source data.

The experimental results for Type D are presented across varying proportions of labeled target-domain data (0.5%, 1%, 2%, 5%, and 10%), enabling a stepwise analysis of performance improvement trends in domain adaptation. Additionally, the “Source-Only” baseline reflects the performance of models trained exclusively on source-domain data (Cityscapes) and evaluated on target-domain images (Foggy Cityscapes). The “Oracle” represents the upper-bound performance obtained by training on fully labeled target-domain data. In Table 2 the highest performance in each category is both highlighted in bold and underlined, the second-highest is in bold, and the third-highest is underlined, allowing for clear visual comparison of results.

Table 2 shows that the YOLOv7-based-source-only model achieves an mAP@0.5 of 50.9, while the Oracle model reaches 68.9, demonstrating strong baseline capability and highlighting the performance ceiling when full supervision is available.

The proposed Type D method outperforms all other method types (A–C) even with just 2% labeled target data. At the 5% label ratio, Type D achieves over 0.4% higher mAP than Type B methods (which use labeled source and unlabeled target data) and more than 8.3% higher mAP than Type C methods (which rely solely on unlabeled target data).

At 10% labeling, Type D delivers further gains—over 1.4% higher mAP@0.5 than Type B, and more than 9.3% higher mAP@0.5 than Type C—while also achieving the best performance across all object categories. Furthermore, the proposed method yields comparable or superior results to GCHQ [17], the best-performing Type B model, further validating its effectiveness. In terms of per-class AP, the proposed approach consistently outperforms existing methods across most object categories, demonstrating not only general performance improvements but also robustness in handling class diversity.

These findings quantitatively confirm that the proposed source-free semi-supervised learning framework is highly effective for domain-adaptive object detection, even under limited label availability, while offering a practical solution that does not require access to source data.

### 4.3. Results on the Sim10k to Cityscapes Dataset

In this experiment, we evaluated the domain adaptation performance from Sim10k (source domain) to Cityscapes (target domain) using the proposed source-free semi-supervised domain adaptation method. The proportion of labeled data in the target domain was set to 0.5%, 1%, 2%, 5%, and 10% in order to analyze performance changes with respect to label availability.

The experimental results are summarized in Table 3 where the best performance on the target domain (Cityscapes) is marked in bold and the best performance on the source domain (Sim10k) is marked in underline. The results in Table 3 indicate that as the ratio of labeled target data increases, the performance on the target domain improves steadily. For example, the mAP@0.5 on the target domain increased from 72.0 at 0.5% labeling to 77.9 at 10% labeling, showing a 5.9-point improvement. In contrast, the performance on the source domain showed a slight decline, but the amount of performance degradation was relatively small compared to the improvements in the target domain. Notably, between 2% and 5%, the source-domain performance either slightly declined or remained stable, suggesting that the proposed method minimizes information loss from the source domain while adapting effectively to the target domain. These findings imply that the proposed learning method not only ensures effective adaptation to the target domain, but also maintains generalization performance across domains, which is a key advantage for building practical domain-adaptive object detection systems.

**Figure 10:**
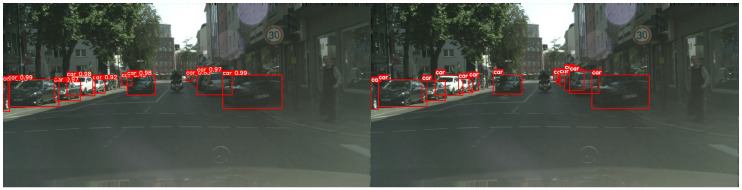
Sim10k→Cityscapes before adaptation(source-only). (**Left**) Prediction on Cityscapes; (**Right**) Ground truth (GT).

Figure 10 provides a visual comparison between the object detection results before domain adaptation and the ground-truth (GT) annotations in the target domain. The left image shows the detection results of a model trained only on the source domain (Sim10k), while the right image shows the corresponding GT annotations for the same scene. Without domain adaptation, some objects are either missed or detected with low accuracy.

**Figure 11:**
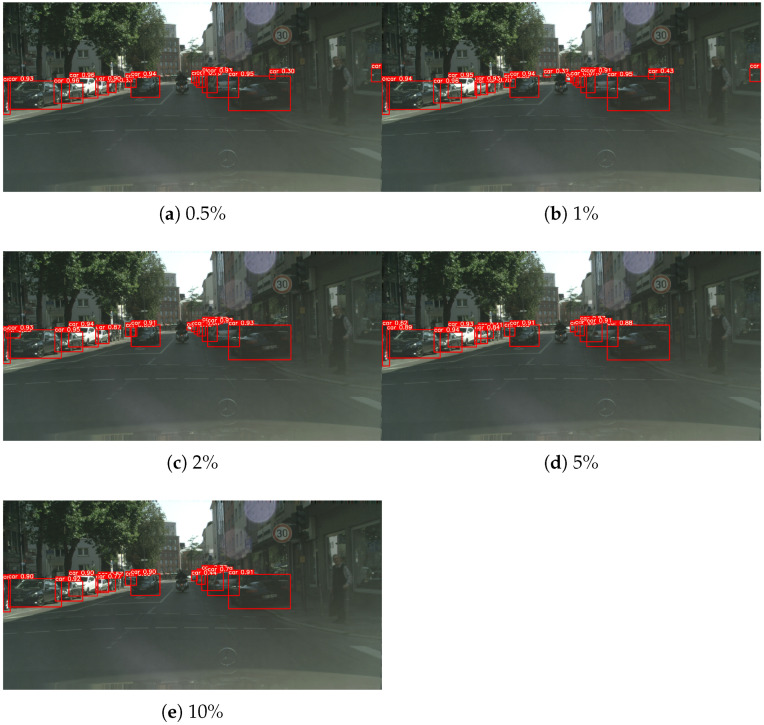
Sim10k→Cityscapes results after adaptation at different labeled target ratios.

Figure 11 presents the detection results on the Sim10k to Cityscapes adaptation setting after applying the proposed method. From top to bottom, the figure shows results from models trained using 0.5%, 1%, 2%, 5%, and 10% labeled target data, which clearly indicate that increasing the proportion of labeled target data strengthens the adaptation effect: false detections are progressively reduced while previously missed objects become correctly detected, leading to more accurate and reliable predictions overall.

Table 4 provides a comparative performance analysis of the proposed method against existing domain adaptation approaches from Sim10k to Cityscapes. All methods are evaluated by AP for the car class, and categorized by training strategy (Types A–D, as in Table 2). For ease of comparison, the best result is marked in bold and underlined, the second-best in bold, and the third-best is underlined for ease of comparison.

As shown, the proposed method (Type D) outperforms most existing methods even with just 0.5% labeled target data, achieving an AP of 72.0. This is notably higher than many earlier approaches that relied on labeled source data. When the label ratio reached 10%, the AP rose to 77.9, which is 8.1 points higher than the best-performing source-free unsupervised method (SF-YOLO, AP = 69.8, Type C).

Furthermore, the proposed method substantially outperforms semi-supervised methods that utilize source data, such as Probabilistic Teacher (PT) [18] with an AP of 55.1 and ConfMix [20] with an AP of 56.3, underscoring the effectiveness of the proposed source-free approach and establishing a new performance benchmark for domain-adaptive object detection in real-world scenarios.

### 4.4. Results on the CCTV Image Dataset

**Figure 12:**
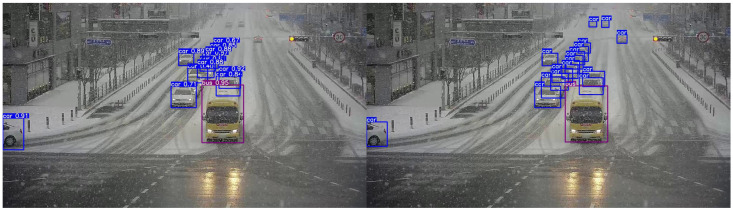
CCTV Clear→Snow before adaptation (source-only). (**Left**) Prediction on snow; (**Right**) Ground truth (GT).

**Figure 13:**
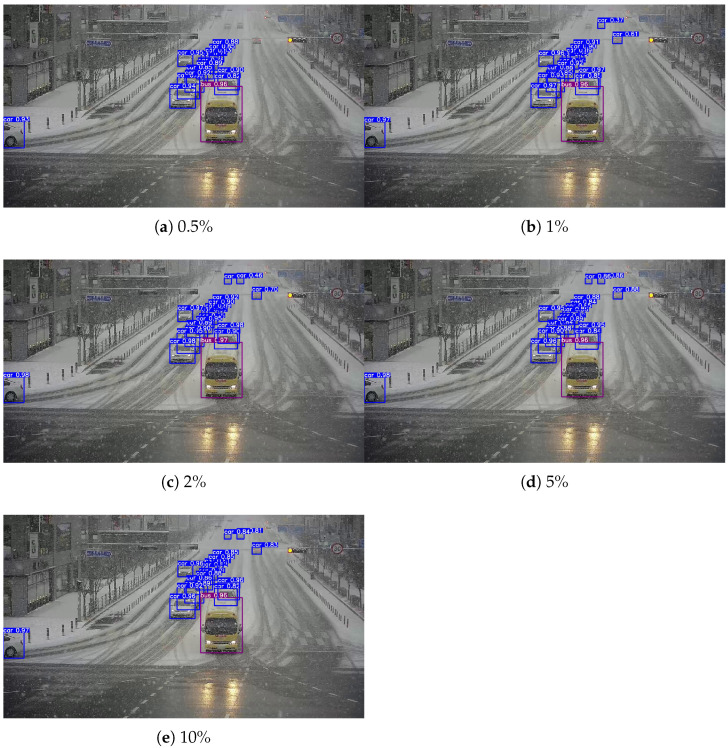
Clear→Snow results on the CCTV image dataset after adaptation at different labeling ratios.

In this experiment, we evaluated the proposed method’s performance for domain adaptation from clear daytime weather to snowy weather using the CCTV image dataset. The labeled data in the target domain (snowy weather) were set at 0.5%, 1%, 2%, 5%, and 10% of the total, and separate training was conducted for each label ratio. Figure 12 and Figure 13 illustrate the results of the domain adaptation experiment. Figure 12 visually compares the object detection results before domain adaptation with the ground-truth annotations in the target domain. The left image shows the detection results from a model trained only on the source domain (clear weather), while the right image shows the corresponding ground-truth annotations for the same scene. Without domain adaptation, some objects were either undetected or detected with low accuracy. Figure 13 presents the detection results after applying the proposed domain adaptation method. From top to bottom, the figure shows results from models trained using 0.5%, 1%, 2%, 5%, and 10% labeled target data. The results clearly demonstrate that as the proportion of labeled target data increases, domain adaptation effectiveness improves: previously undetected objects were identified, and the confidence scores of detected objects improved overall.

Table 5 summarizes the quantitative performance metrics for the domain adaptation experiment, comparing clear- to snowy-weather conditions. The proposed source-free domain adaptation method was applied with 0.5%, 1%, 2%, 5%, and 10% of labeled data from the target domain (snowy weather). The best performance on the target domain is indicated in bold, while the best performance on the source domain is underlined. while the best performance on the source domain is underlined.

The results in Table 5 highlight several key findings:

- Target-Domain Performance: The overall performance on the target domain (snowy weather) significantly improved compared to the pre-adaptation baseline.
  · For instance, mAP@0.5 increased from 78.4 (before adaptation) to 86.3 when using 10% labeled data, showing a 7.9% improvement.
  · Recall also consistently increased with the labeling ratio, with a maximum gain of +10.7 percentage points.
- Robust Domain Adaptation: Despite the relatively small overall dataset size, the proposed method demonstrated robust domain adaptation capability.
  · Notably, performance improvements were evident even with just 0.5–5% labeled data.
  · Although performance on the source domain slightly declined, the magnitude of this decline was minimal compared to the improvements observed in the target domain.
- Best Performance with 10% Labeled Data: With 10% labeled target data, the model achieved the highest performance across all metrics in the target domain.
  · These results strongly support the practical applicability of the proposed framework, particularly in real-world settings like real-time CCTV environments.

### 4.5. Ablation Study

This section presents an ablation study to analyze the impact of each component of the proposed source-free semi-supervised learning method. The experiments were conducted using the Cityscapes and Foggy Cityscapes datasets to investigate how removing or altering individual components affects performance. By comparing results with and without specific modules, or using alternative strategies, we aim to quantitatively demonstrate the effectiveness of the proposed framework.

#### 4.5.1. Ablation of Static Adversarial Regularization

In our method, static adversarial regularization (SAR) is employed to improve domain generalization performance. This approach acts as a stationary regularizer, using a fixed discriminator to suppress the model’s overfitting to domain-specific artifacts and guiding the model to learn more generalized, domain-invariant features. To validate this approach, we conducted an ablation study, comparing three settings under identical training protocols, and Table 6 summarizes the performance of these configurations.

##### Baselines and Variants

- Baseline (without SAR): In both the source pre-training and target training stages, no domain-adversarial learning is applied. During source pre-training, the model is optimized using only the supervised loss, while in the target training stage, the parameters are updated solely based on the supervised loss, consistency loss, and distillation loss.
- Dynamic Adversarial Ablation (DANN-style, SFDA adaptation): This comparative method employs DANN-style dynamic adversarial learning adapted for the SFDA setting, leveraging a trainable domain discriminator (D) and a gradient reversal layer (GRL). During the target adaptation stage, D is trained to predict the target (1), and the backbone is updated via the GRL to appear as source (0), thereby forming a minimax game.
  DANN-style (Source: Minimax, Target: Minimax)
  · Stage 1 —Source Pre-training: Perform minimax optimization using source data only. D is optimized to predict source (0), and the backbone is driven (via the GRL) to fool D toward target (1).
  · Stage 2 —Target Adaptation: Perform minimax optimization using target data only. D is optimized to predict target (1), and the backbone is driven (via the GRL) to fool D toward source (0).
- Our Method (with SAR): The proposed setting, which uses a fixed, static discriminator as a stationary regularizer to suppress overfitting to source-specific artifacts.

**Figure 14:**
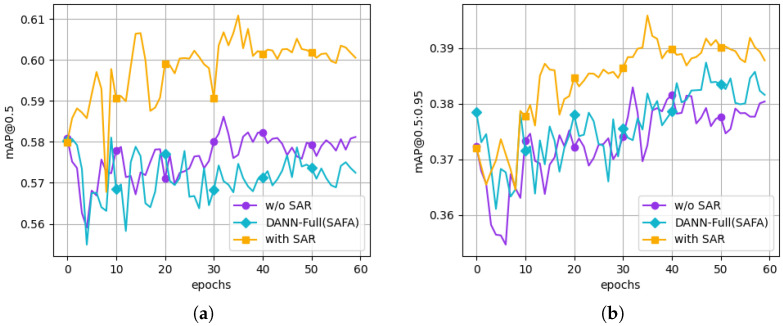
Ablation plots comparing SAR with dynamic adversarial training (DANN-style) under SFDA. Target-domain validation performance over epochs: (**a**) mAP@0.5 and (**b**) mAP@0.5:0.95 for w/o SAR, DANN-style, and with SAR. All curves are shown without smoothing.

As shown in Figure 14, SAR exhibits more stable convergence and reaches a higher validation mAP trend than the variant without the SAR baseline and the DANN-style variant, achieving the best scores on all metrics except recall, leading to a more reliable and robust detection performance overall. The results for the DANN-style variant suggest that running a minimax game in both stages can over-confuse representations and harm localization quality. Overall, by combining static discriminator with a GRL, SAR provides a stable regularization signal that reduces false positives without training oscillation and improves box quality. These findings offer empirical evidence that SAR substantially enhances detection quality and convergence stability in domain-invariant learning.

#### 4.5.2. Ablation of Pseudo-Label Fusion

**Figure 15:**
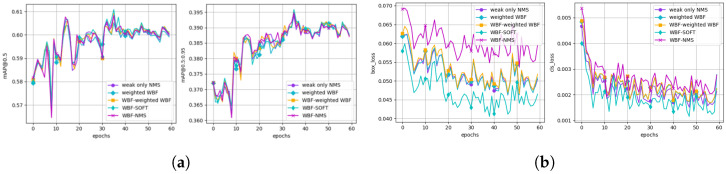
Ablation of pseudo-label fusion strategies.Although the differences are marginal, WBF-SOFT achieves the best overall scores (see Table 7). (**a**) Target validation performance over epochs (mAP@0.5 and mAP@0.5:0.95). (**b**) Training loss curves over epochs (box loss and classification loss).

While previous studies typically generate pseudo-labels using only weak augmentations in the teacher model, we propose a fusion strategy that leverages both weak and strong augmentations. The corresponding predictions are then combined using weighted box fusion (WBF) and Soft-NMS to generate more accurate and stable pseudo-labels. Table 7 presents a comparison of different pseudo-label fusion strategies, with the best performance in each metric highlighted in bold. To complement these endpoint results, Figure 15 visualizes the training dynamics: Figure 15a reports target validation mAP (mAP@0.5 and mAP@0.5:0.95), and Figure 15b shows the corresponding training losses (box and classification). Although the differences are marginal, WBF-SOFT consistently attains the best overall scores, aligning with the quantitative results in Table 7.

The experimental results show that processing each augmented prediction with WBF followed by fusion using Soft-NMS yields the best performance. In particular, WBF-SOFT improves mAP@0.5 from 60.3 (weighted WBF) to 60.7 (+0.4) and precision from 82.9 to 83.7 (+0.8), which quantifies the incremental contribution of Soft-NMS on top of WBF. In addition, WBF-SOFT outperforms WBF-NMS (mAP@0.5=59.8), indicating that Soft-NMS is more effective than hard NMS at handling duplicate boxes while preserving valid pseudo-labels. Overall, these results both suggest that pseudo-label quality has a significant impact on overall training performance and empirically validate the effectiveness of the proposed pseudo-label fusion method.

#### 4.5.3. Ablation of Labeled and Unlabeled Data Weight Adjustment

In our method, a time-varying (epoch-scheduled) exponential weighting balances the contributions of labeled and unlabeled data during training, effectively controlling the influence of labeled data. To evaluate the effect of different weighting strategies, we compare three approaches: static, linear, and exponential. These methods aim to control the influence of supervised and unsupervised data throughout training.

- Static: In this method, fixed weights α and β are applied to the losses from labeled and unlabeled data, respectively, maintaining a constant contribution throughout the training process.
- Linear: In this method, the weight for labeled data linearly decreases from an initial value $\alpha_{start}$ to a final value $\alpha_{end}$, while the weight for unlabeled data increases linearly from $\beta_{start}$ to $\beta_{end}$ as training progresses.
- Exponential: This method uses the same starting and ending values as the linear approach but adjusts the weights exponentially based on the training stage.

Table 8 presents the experimental results for these three weighting strategies, with the best performance in each metric highlighted in bold. As shown in the table, the exponential weighting scheme achieves the best performance in both precision and mAP metrics, demonstrating that gradually adjusting the contributions of labeled and unlabeled data over time is effective for improving training stability and overall model performance.

## 5. Discussion

- Pseudo-Label Fusion + Exponential Weighting: We learn pseudo-labels via weak-to-strong augmentation consistency and apply a time-varying exponential weighting (small at the beginning, gradually increased) to suppress early noise propagation. This follows the consistency-pseudo-labeling paradigm of FixMatch-style methods [41] and the weighting/EMA practice of Mean Teacher approaches. Importantly, the proposed components are motivated by commonly observed challenges in source-free semi-supervised adaptation, including pseudo-label noise accumulation and the difficulty in balancing labeled and unlabeled training signals under domain shift.
- Static Adversarial Regularization (SAR): Using a static discriminator (a fixed D) with a GRL as an auxiliary regularizer preserves DANN’s principle of learning domain-invariant features via domain confusion, while reducing the oscillation and instability of inherent in simultaneous min–max optimization.
- Limitations ( training setup sensitivity and practical robustness): We acknowledge that source-free semi-supervised adaptation can be sensitive to training setup because unlabeled learning relies on imperfect pseudo-labels and labeled target supervision is limited. Our goal is not to claim universal optimality across all detectors and domain shifts, but to provide a practical and stable source-free semi-supervised adaptation recipe under common surveillance constraints. While a large-scale hyperparameter sweep is beyond the scope of this work, we emphasize that our key settings were chosen within ranges commonly recommended in teacher–student learning and the recent detection-based adaptation literature. In particular, high EMA values (0.99–0.999x) are widely used to stabilize the teacher, and conservative pseudo-label filtering is a standard practice to suppress false positives under label noise. Consistent with these established practices, we adopt conservative learning rate and pseudo-label thresholds to reduce error accumulation during early adaptation, and we adjust EMA depending on the data scale to balance teacher stability with adaptation lag. A systematic robustness study under controlled hyperparameter perturbations (EMA, learning rate, and pseudo-label thresholds) is an important future direction.
- Future work: Building on the observed training setup sensitivity, we will pursue (i) adaptive scheduling of EMA and pseudo-label thresholds based on teacher confidence/calibration signals, (ii) uncertainty-aware pseudo-label refinement to reduce noise accumulation, and (iii) a controlled robustness evaluation where EMA, adaptation learning rate, and confidence thresholds are perturbed within predefined ranges and stability is quantified using metrics such as the variance of validation mAP and the rate of performance degradation across epochs. We will also extend evaluation to broader CCTV shifts (illumination, viewpoint, compression) and investigate continual/online adaptation for long-term deployment. In addition, we will consider more realistic surveillance degradations beyond domain shift, including general image corruptions and compression/re-encoding artifacts commonly observed in practical pipelines [42,43].

## 6. Conclusions

In this study, we proposed a domain-adaptive, source-free semi-supervised learning framework for effective object detection in the target domain without requiring access to source data. Building on the Mean Teacher framework, we designed a training architecture that enhances both stability and performance. This architecture combines pseudo-label fusion using weak and strong augmentations, static adversarial regularization, which effectively guides the model toward domain-invariant features, and a time-varying (epoch-scheduled) exponential weighting that adjust the influence of labeled and unlabeled data. The proposed approach was evaluated across multiple domain adaptation scenarios: Cityscapes → Foggy Cityscapes, Sim10k → Cityscapes, and Clear → Snowy weather (CCTV dataset). The experimental results show that our method outperforms other representative domain adaptation and source-free techniques. Notably, even with just 2% of labeled target data, our method achieves up to a 7.9% performance improvement compared to existing semi-supervised and source-free methods, underscoring the effectiveness of both the static adversarial regularization and pseudo-label fusion components.

Additionally, to address training instability and prevent overfitting to the target domain, we introduced a progressive weighting mechanism that dynamically adjusts the contributions of labeled and unlabeled data throughout training. This strategy enables steady performance improvements in the target domain, while minimizing the drop in source-domain generalization. Our work provides a practical solution for real-time object detection tasks in domain adaptation settings, particularly in CCTV-based video analysis systems, where the reuse of source data may be restricted due to privacy concerns. In future work, we plan to explore uncertainty-based pseudo-label selection, techniques to address class imbalance, and extensions to multi-domain adaptation scenarios.

Despite the consistent gains, the proposed framework may degrade under extreme target shifts (e.g., nighttime illumination, strong motion blur, or compression artifacts) or when pseudo-label noise accumulates under severe class imbalance and heavy occlusion, which can be critical in CCTV scenarios with small and distant objects. The adaptation process can also be sensitive to key training choices (EMA rate, adaptation learning rate, and pseudo-label thresholds), and a large-scale hyperparameter robustness study is beyond the scope of this work.

Future work will focus on uncertainty-aware pseudo-label refinement and the adaptive scheduling of EMA and pseudo-label thresholds based on teacher confidence to reduce noise accumulation and setup sensitivity. We also plan a controlled robustness evaluation under bounded hyperparameter perturbations and broader CCTV shifts, including continual/online adaptation for long-term deployment and strategies to better handle class imbalance and multi-domain adaptation scenarios.

## Figures and Tables

**Table 1:** Domain adaptation results on the Cityscapes → Foggy Cityscapes dataset (under varying label ratios).

Label Ratio	Domain	Precision	Recall	mAP@0.5	mAP@0.5:0.95
Source-only	Cityscapes	84.6	61.9	71.2	45.6
	Foggy Cityscapes	73.8	45.9	50.9	33.0
0.5%	Cityscapes	76.9 (↓7.7)	55.7 ( ↓ 6.2)	63.3 (↓7.9)	38.6 (↓7.0)
	Foggy Cityscapes	78.2 (↑4.4)	52.4 (↑6.5)	58.6 (↑7.7)	36.3 (↑3.3)
1%	Cityscapes	75.8 (↓8.8)	58.4 (↓3.5)	64.3 (↓6.9)	39.4 (↓6.2)
	Foggy Cityscapes	80.0 (↑6.2)	53.7 (↑7.8)	58.9 (↑8.0)	36.1 (↑3.1)
2%	Cityscapes	79.4 (↓5.2)	53.9 (↓8.0)	61.3 (↓9.9)	36.9 (↓8.7)
	Foggy Cityscapes	82.8 (↑9.0)	52.6 (↑6.7)	59.5 (↑8.6)	37.1 (↑4.1)
5%	Cityscapes	79.1 (↓5.5)	58.2 (↓3.7)	63.9 (↓7.3)	39.6 (↓6.0)
	Foggy Cityscapes	82.3 (↑8.5)	**54.0** (↑8.1)	59.9 (↑9.0)	38.3 (↑5.3)
10%	Cityscapes	79.7 (↓4.9)	56.1 (↓5.8)	63.6 (↓7.6)	40.5 (↓5.1)
	Foggy Cityscapes	**83.1** (↑9.3)	53.0 (↑7.1)	**60.9** (↑10.0)	**39.4** (↑6.4)

**Note:** The arrows ↑ and ↓ indicate performance increase and decrease relative to the baseline, respectively. For each metric, the best result on the target domain is shown in bold, and the best result on the source domain is underlined.

**Table 2:** Comparative performance on Cityscapes to Foggy Cityscapes domain adaptation. ‘Oracle’ refers to the model trained with 100% labeled target data using the same architecture (YOLOv7) and training protocol, representing the supervised upper bound of performance.

Type	Method	Detector	Source	Target	Bus	Bicycle	Car	Mcycle	Person	Rider	Train	Truck	mAP
A	PICA [24]	Faster-RCNN	✓	few-shot	28.1	33.7	43.0	25.4	28.3	41.3	24.3	23.8	32.2
	PT [18]	Faster-RCNN	✓	×	51.8	44.5	59.7	35.4	40.2	48.8	30.6	30.7	42.7
	AT [19]	Faster-RCNN	✓	×	56.3	51.9	64.2	38.5	45.5	55.1	54.3	35.0	50.9
B	CMT [15]	Faster-RCNN	✓	×	**66.0**	51.2	63.7	41.4	45.9	55.7	38.8	39.6	50.3
	ConfMix [20]	YOLOv5s	✓	×	45.8	33.5	62.6	28.6	45.0	43.4	40.0	27.3	40.8
	SSDA-YOLO [16]	YOLOv5l	✓	×	63.0	53.6	74.3	47.4	60.6	62.1	48.0	37.8	55.9
	GCHQ [17]	YOLOv7	✓	×	64.7	55.8	77.5	48.9	60.1	63.1	**58.9**	**47.1**	59.5
C	LODS [8]	Faster-RCNN	×	×	39.7	37.8	48.8	33.2	34.0	45.7	19.6	27.3	35.8
	IRG [25]	Faster-RCNN	×	×	39.6	41.6	51.9	31.5	37.4	45.2	25.2	24.4	37.1
	MemCLR [33]	Faster-RCNN	×	×	40.6	42.2	52.4	29.4	37.7	42.8	31.7	24.5	37.7
	PETS [21]	Faster-RCNN	×	×	39.3	41.6	56.3	34.2	42.0	48.7	5.5	19.3	35.9
	DRU [22]	Deformable DETR	×	×	43.2	48.6	62.5	34.2	48.3	51.5	34.1	26.2	43.6
	SF-YOLO [23]	YOLOv5l	×	×	53.7	50.5	71.5	40.6	55.5	58.0	46.1	36.6	51.6
D	Source only	Faster-RCNN	·	·	27.5	32.6	37.3	24.2	34.9	42.2	5.0	13.8	27.2
		Deformable DETR	·	·	24.3	37.7	46.2	19.0	37.3	43.6	5.2	9.2	27.8
		YOLOv7	·	·	54.7	54.8	65.5	47.3	58.5	62.1	34.1	30.3	50.9
	Proposed	YOLOv7	×	0.5%	64.2	55.0	75.3	52.1	62.3	64.7	54.4	40.7	58.6
			×	1%	64.8	57.3	76.5	51.1	63.1	65.1	54.9	38.8	58.9
			×	2%	65.1	56.4	77.4	**53.8**	63.8	**66.3**	55.4	37.8	59.5
			×	5%	**67.1**	**58.2**	**78.3**	**53.8**	**65.5**	65.6	53.1	37.4	**59.9**
			×	10%	**66.0**	**59.0**	**78.8**	**53.0**	**65.3**	**66.2**	**56.8**	**41.7**	**60.9**
	Oracle	YOLOv7	·	·	76.4	64.7	84.0	62.0	73.0	72.6	65.3	53.4	68.9

**Note:** ✓ indicates used, × indicates unused, and · indicates not applicable. For each metric, the best, second-best, and third-best results are indicated by bold, bold underlined, and underlined text, respectively. **Fairness note:** To minimize architectural confounds, Type D results (and the “Source-only/Oracle” baselines) are evaluated using the same YOLOv7 implementation and training procedure; prior works are reported from the original papers with detector architectures explicitly indicated in the “Detector” column.

**Table 3:** Domain adaptation results on the Sim10k → Cityscapes dataset (under varying label ratios).

Label Ratio	Domain	Precision	Recall	mAP@0.5	mAP@0.5:0.95
Source-only	Sim10k	94.3	88.5	94.2	72.6
	Cityscapes	81.5	58.4	61.1	41.1
0.5%	Sim10k	92.6 ( ↓1.7)	83.5 (↓5.2)	90.3 (↓3.9)	61.0 (↓11.6)
	Cityscapes	82.5 (↑1.0)	66.1 (↑7.7)	72.0 (↑10.9)	50.1 (↑9.0)
1%	Sim10k	91.5 (↓2.8)	83.3 (↓5.4)	89.9 (↓4.3)	59.6 (↓13.0)
	Cityscapes	83.3 (↑1.8)	66.3 (↑7.9)	72.1 (↑10.8)	50.4 (↑9.3)
2%	Sim10k	91.0 (↓3.3)	84.6 (↓3.9)	90.4 (↓3.8)	61.0 (↓11.6)
	Cityscapes	**85.2** (↑3.7)	68.6 (↑10.1)	75.1 (↑14.0)	52.7 (↑11.6)
5%	Sim10k	91.9 (↓2.4)	84.0 (↓4.5)	91.0 (↓3.2)	60.9 (↓11.7)
	Cityscapes	84.2 (↑2.7)	70.0 (↑11.6)	75.9 (↑14.8)	54.2 (↑13.1)
10%	Sim10k	91.9 (↓2.4)	82.1 (↓6.4)	90.3 (↓3.9)	59.4 (↓13.2)
	Cityscapes	83.6 (↑2.1)	**71.5** (↑13.1)	**77.9** (↑16.8)	**56.5** (↑15.4)

**Note:** The arrows ↑ and ↓ indicate an increase and a decrease in performance relative to the baseline, respectively. For each metric, the best result in the target domain is shown in bold, and the best result in the source domain is underlined.

**Table 4:** Comparative Performance on Sim10k to Cityscapes domain adaptation. ‘Oracle’ refers to the model trained with 100% labeled target data using the same architecture (YOLOv7) and training protocol, representing the supervised upper bound of performance.

Type	Method	Detector	Source	Target	AP (car)
A	PICA [24]	Faster-RCNN	✓	few-shot	42.1
B	PT [18]	Faster-RCNN	✓	×	55.1
	ConfMix [20]	YOLOv5s	✓	×	56.3
C	IRG [25]	Faster-RCNN	×	×	43.2
	MemCLR [33]	Faster-RCNN	×	×	44.2
	PETS [21]	Faster-RCNN	×	×	57.8
	DRU [22]	Deformable DETR	×	×	58.7
	SF-YOLO [23]	YOLOv5l	×	×	69.8
D	Source only	YOLOv7	·	·	61.1
	Proposed	YOLOv7	×	0.5%	72.0
			×	1%	72.1
			×	2%	75.1
			×	5%	**75.9**
			×	10%	**77.9**
	Oracle	YOLOv7	·	·	84.3

**Note:** ✓ indicates used, × indicates unused, and · indicates not applicable. For each metric, the best result is shown in bold, the second-best result is shown in bold and underlined, and the third-best result is underlined.

**Table 5:** Domain adaptation results on the Clear → Snowy CCTV dataset (under varying label ratios).

Label Ratio	Domain	Precision	Recall	mAP@0.5	mAP@0.5:0.95
Source-only	Clear	94.2	91.2	95.1	80.3
	Snow	75.5	78.0	78.4	56.4
0.5%	Clear	94.0 ( ↓ 0.2)	89.1 (↓2.1)	94.6 (↓0.5)	78.0 (↓2.3)
	Snow	75.9 (↑0.4)	79.3 (↑1.3)	80.9 (↑2.5)	55.0 (↓1.4)
1%	Clear	95.7 (↑1.5)	86.2 (↓5.0)	93.5 (↓1.6)	75.9 (↓4.4)
	Snow	60.9 (↓14.6)	**88.7** (↑10.7)	84.8 (↑5.9)	54.3 (↓2.1)
2%	Clear	91.2 (↓3.0)	89.4 (↓1.8)	93.6 (↓1.6)	74.3 (↓6.0)
	Snow	**79.6** (↑4.1)	84.1 (↑6.1)	83.3 (↑4.9)	56.5 (↑0.1)
5%	Clear	96.4 (↑2.2)	86.5 (↓4.7)	94.0 (↓1.1)	74.9 (↓5.4)
	Snow	69.5 (↓6.0)	88.5 (↑10.5)	84.7 (↑6.3)	56.5 (↑0.1)
10%	Clear	96.6 (↑2.4)	85.5 (↓5.7)	93.5 (↓1.6)	76.3 (↓4.0)
	Snow	72.2 (↓3.3)	86.5 (↑8.5)	**86.3** (↑7.9)	**59.0** (↑2.2)

**Note:** The arrows ↑ and ↓ indicate an increase and a decrease in performance relative to the baseline, respectively. For each metric, the best result in the target domain is shown in bold, and the best result in the source domain is underlined.

**Table 6:** Performance comparison across SAR and DANN under SFDA.

Setting	P	R	mAP@0.5	mAP@0.5:0.95
w/o SAR	80.0	51.7	58.1	38.0
DANN-style (SFDA)	81.9	51.9	58.0	37.7
with SAR	**84.1**	**52.7**	**60.9**	**39.5**

**Note:** For each metric, the best result is shown in bold.

**Table 7:** Comparison of different pseudo-label fusion strategies.

Setting	P	R	mAP@0.5	mAP@0.5:0.95
weak only NMS	79.7	53.4	60.0	**39.6**
weighted WBF	82.9	51.3	60.3	39.5
WBF–weighted WBF	78.5	**53.5**	60.5	39.4
WBF–SOFT	**83.7**	51.3	**60.7**	**39.6**
WBF–NMS	82.8	53.3	59.8	**39.6**

**Note:** For each metric, the best result is shown in bold.

**Table 8:** Comparison of different weight adjustment strategies.

Setting	P	R	mAP@0.5	mAP@0.5:0.95
Static	83.4	51.9	59.9	39.0
Linear	81.1	**53.5**	59.7	**39.6**
Exponential	**83.7**	51.3	**60.7**	**39.6**

**Note:** For each metric, the best result is shown in bold.

## Data Availability

Publicly available datasets were analyzed in this study, including Cityscapes (https://www.cityscapes-dataset.com, accessed on 10 December 2025) and Sim10k (https://fcav.engin.umich.edu/projects/driving-in-the-matrix, accessed on 10 December 2025). In addition, this study used the CCTV traffic video dataset (Urban Roads) from “The Open AI Dataset Project (AI-Hub, Republic of Korea)” (AI-Hub: https://www.aihub.or.kr, accessed on 10 December 2025). Due to national data policies, access to the AI-Hub dataset is restricted to users affiliated with domestic institutions in the Republic of Korea.

## Abbreviations

The following abbreviations are used in this manuscript:

DA	Domain Adaptation
DAOD	Domain Adaptive Object Detection
SAR	Static Adversarial Regularization
GRL	Gradient Reversal Layer
EMA	Exponential Moving Average
DANN	Domain Adversarial Neural Network
NMS	Non-Maximum Suppression
WBF	Weighted Box Fusion
